# Cascading Effects of Cover Crops on the Subsequent Cash Crop Defense against the Polyphagous Herbivore Fall Armyworm (*Spodoptera frugiperda*)

**DOI:** 10.3390/insects14020177

**Published:** 2023-02-10

**Authors:** Adegboyega Fajemisin, Alexis Racelis, Rupesh Kariyat

**Affiliations:** 1 School of Earth Environmental and Marine Sciences, University of Texas Rio Grande Valley, Edinburg, TX 78539, USA; 2 Department of Entomology and Plant Pathology, University of Arkansas, Fayetteville, AR 72701, USA

**Keywords:** *Spodoptera frugiperda*, plant soil-feedbacks, cover crops, herbivore resistance

## Abstract

**Simple Summary:**

Although studies have started to show that the effects of cover crops can translate into the cash cropping season, there is little information on the cascading effects of cover crops on the subsequent cash crop defenses, especially against polyphagous herbivores. To bridge this information gap, we conducted a field and laboratory study to investigate the cascading effects of different cover crop species on the subsequent cash crop defense against the polyphagous herbivore fall armyworm (*Spodoptera frugiperda*) across three fields in the Lower Rio Grande Valley in south Texas. Our field and laboratory results revealed that cover crop treatments differentially affected *S. frugiperda* growth and development. We also show that cover crops had a positive cascading effect on the growth and development of *S. frugiperda* on the subsequent cash crop. Collectively, our results add an additional line of evidence on how cover crops can affect pest dynamics outside the cash crop season.

**Abstract:**

Recent studies have started to show that the benefits of cover crops can cascade to the cash crop growing seasons. However, the impact of cover crops on the subsequent cash crop defense against herbivores is not well understood. To test this, we conducted a field and laboratory study to assess the possible cascading effects of cover crops such as *Vigna unguiculata*, *Sorghum drummondii*, *Raphanus sativus*, and *Crotalaria juncea* on the subsequent cash crop (*Sorghum bicolor*) defense against the notorious polyphagous herbivore fall armyworm (*Spodoptera frugiperda*) across three farms in the Lower Rio Grande Valley. Our field and laboratory studies showed that the cash crop planted in the cover crop treatment differentially affected *S. frugiperda*. More specifically, we found that cover crops have positive effects on the growth and development of *S. frugiperda* on the subsequent cash crop, including both larval and pupal parameters. However, our experiments on physical and chemical defenses in cash crops failed to show any significant differences between cover and control. Collectively, our results add an additional line of evidence on the effects of cover crops on pest dynamics outside the cash crop season, a key consideration for the selection and management of cover crops and cash crops, whose underlying mechanisms need to be examined further.

## 1. Introduction

Cover cropping is one of the oldest farming practices and is currently getting national and worldwide attention [1]. According to the US Agriculture Census, over 6.2 million hectares were planted with cover crops in 2017 (USDA-NASS). Cover crops are non-harvested crops that are cultivated in addition to the main cash crop to enhance ecosystem services such as soil fertility, carbon sequestration, the attraction of pollinators, soil water availability, weed management, and pest management [2,3,4]. The benefits of cover crops are commonly estimated during the cover cropping season [4,5], and include, but are not limited to weed suppression [6], reduction of soil erosion [7,8], enhancement of soil organic matter [9,10], and even pest management [3,4]. Recent studies have also started to show that some of these effects, especially in pest management, can cascade from cover to the cash crop growing seasons [11,12,13].

A major concern about cover cropping is soil moisture loss and moisture availability to the subsequent cash crops [14,15]. This is more common in semi-arid and dryland regions where non-irrigated cropping systems are common [14,16]. Studies have shown that cover crops can negatively impact soil water content and nutrients, thereby significantly impacting the yield of the subsequent cash crops. For example, [17] an experiment conducted in Kansas demonstrated that cover crops significantly reduced the yield of the subsequent cash crop by 31%. Similarly, [14] showed that cover crop legacy effects could be detrimental to the yield of the subsequent cash crop. Interestingly, cover crops, in combination with other abiotic factors, such as soil water content and temperature, have been shown to impact arthropod populations, especially pests of economic importance [2,4]. For example, the management of bollworm (*Helicoverpa armigera*) in non-Bt cotton was achieved by increasing the population of predators of *Geocoris punctipes* and fire ants (*Selenopsis invicta*) through the cultivation of the cover crops, crimson clover, and rye [18]. Similarly, [4] showed that cover crops significantly enhanced the population of beneficial arthropods early in the season in an organic farming system in south Texas. Conversely, cover crops have also been found to promote the population of herbivores in the agroecosystem. For example, [19] demonstrated that corn fields grown with rye cover crops significantly harbored more true armyworms (*Mythimna unipuncta* Haworth) with significantly higher damage to corn plants. Clearly, the effects of cover crops on pests and their cascading effects on cash crops needs to be better understood with more regional and species-specific studies.

The fall armyworm (FAW; *Spodoptera frugiperda* J.E. Smith) is a native pest of neotropical areas of Central and South America. In recent years, it has emerged as one of the most important global polyphagous pests of crops [20,21]. FAW is a serious concern in various agroecosystems and can feed on most of the cover and cash crops [22,23]. The management of FAW in recent years has ranged from the use of synthetic pesticides to biopesticides, cultural control, and biological control [20]. However, studies have also shown that cover crops can prime the defense of subsequent cash crops against herbivores [4,11]. For example, [3] showed that arbuscular mycorrhizal fungi (AMF) inoculated cover crops had significantly better fitness and received lower levels of herbivory compared to control plants, more specifically on the incidence and damage by FAW. As FAW is a serious pest of crops that potentially impacts food security, and since they feed on both cover and cash crops across the globe, a better understanding of any possible cascading effects of FAW on cash crop defenses can provide insights into the ecosystem services of cover crops, especially in pest management.

To test this, we designed field and laboratory experiments where we allowed FAW to feed on cash crops grown in soils that had been planted with cover crops in the previous season, followed by measuring FAW growth and development traits. This was followed by a detailed lab experiment where we examined these traits with plant materials added to an artificial diet, and asked at what stages, if any, the effects cascade into. We then followed up these experiments by examining physical (wax, trichomes) and chemical (poly phenol oxidase activity) in the cash crop. We hypothesized that cover crops would have a positive cascading effect on the subsequent cash crop defense against herbivores (*S. frugiperda*), possibly through modified plant–soil feedback that enhances the constitutive defenses in the cash crop [3,11,13].

## 2. Materials and Methods

### 2.1. Study Sites

This study was conducted in three agricultural fields (26°18′12.2″ N 97°50′13.1″ W, 26°24′35.1″ N 98°27′52.8″ W, and 26°20′32.6″ N 98°31′51.6″ W) located in three different counties (Hidalgo, Willacy, and Cameron) in the Lower Rio Grande Valley, Texas. The farms have been used to produce cash crops such as corn, sorghum, sunflower, and cotton, with soil types in each farm ranging from fine sandy loam to sandy clay loam. The average temperature during the summer and fall of 2021 ranged between 30 °C and 27.2 °C, respectively, while the average temperatures during the spring and summer of 2022 ranged between 23.9 °C to 31.7 °C. The farms, locations, and coordinates are detailed in Supplementary Figure S1a–d.

### 2.2. Cover Crop Treatments and Experimental Design

The cover crop treatments used in this study were selected based on their ability to withstand the extremely hot summer conditions of south Texas. The cover crop treatments include (1) sorghum–sudangrass (*Sorghum drummondii*), a grass with high biomass used for suppressing weeds and nematodes and increasing soil organic matter; (2) cowpea (*Vigna unguiculata*), a legume that fixes atmospheric nitrogen with moderate to high potential to suppress weeds and improve soil health; (3) radish (*Raphanus sativus*), a vegetable that provides soil cover, suppresses weeds and has the potential to alleviate soil compaction; and (4) sunn hemp (*Crotalaria juncea*), a legume that reduces soil erosion, conserves soil water and recycles plant nutrients. Our first (Hunter; 26°27′32.4″ N 97°53′15.6″ W) field had a mix of cowpea (*V. unguiculata*) and sorghum–sudangrass (*S. drummondii*). The second field (Cemetery; 26°18′12.2″ N 97°50′13.1″ W) had a mixture of cowpea (*V. unguiculata*), sorghum–sudangrass (*S. drummondii*), and sunn hemp (*C. juncea*) while the third field (Mahac; 26°24′35.1″ N 98°27′52.8″ W) had a mixture of cowpea (*V. unguiculata*) and radish (*R. sativus*). The cover crop seeds were purchased from Hancock seeds (Dade City, FL, USA). After the cash crop growing season, in July 2021, an area of approximately 161.9, 80.9, and 81.0 ha in Mahac, Hunter, and Cemetery, respectively, was disked, followed by the planting of the different cover crop species at a row spacing of 0.19 m.

### 2.3. Planting and Termination

Cover crop seeds were planted in early August 2021 using a front-folding grain drill (John Deere, Moline, IL, USA) at recommended rates (Table 1). The cover crops were terminated in mid-December 2021.

### 2.4. Cash Crop Planting

The cover crops were terminated in mid-December 2021, and the residue biomass was incorporated into the soil. Cash crop seeds (*Sorghum bicolor*) were purchased from Hancock seeds (Dade City, FL, USA). Cash crops were planted using a front-folding grain drill (John Deere, Moline, IL, USA) in the control and cover crop plots in each field at a row spacing of 0.19 m in March 2022 at the recommended rate (Table 2).

### 2.5. Insect Colony

*S. frugiperda* eggs were purchased from a commercial vendor (Frontier Agricultural Services, Calexico, CA, USA). The eggs were globular in shape, and the color appeared pale green for a day after they were received and then turned yellow and black before hatching. The eggs were allowed to hatch at room temperature, after which they were transferred to petri dishes with artificial diets. For more details about FAW rearing, please see [24].

### 2.6. Artificial Diets

*S. frugiperda* caterpillars were allowed to feed on a wheat germ-based artificial diet, which was prepared based on the recommendations of the commercial vendor (Frontier Agricultural Sciences): 100 mL of water was boiled to 85 °C followed by adding 250 g of the artificial diet, which was then thoroughly mixed until there were no more lumps. For detailed information about the preparation of artificial diets, see [24,25].

### 2.7. Field Experimental Design

A total of 144 third-instar *S. frugiperda* larvae were used to assess the effect of cover crops on the subsequent cash crop defense against herbivores across three fields. The fields were sectioned into control and cover crop treatment plots. In each plot (control and cover) across the fields, 24 *S. frugiperda* larvae were preweighed and enclosed in a mesh with fully opened young sorghum (cash crop) leaves. After three days, the *S. frugiperda* larvae were collected and weighed in the lab to evaluate mass gain. The caterpillars were then allowed to feed on an artificial diet until they pupated [23,24].

### 2.8. Laboratory Experimental Design

Sorghum newest leaves (50 g) were collected from the control and cover treatment plots across the three fields. One leaf was collected per plant totaling 24 leaves in each treatment plot (control and cover crop) across the three fields. They were subjected to cryo-drying, followed by grinding the leaves with a mortar and pestle to a fine powder, after which they were added to 0.5 L of artificial diet. A total of 180 third instar *S. frugiperda* larvae were used to assess the effect of cover crops on the subsequent cash crops. For each treatment, preweighed caterpillars (n = 30; second instar) were allowed to feed on diets, and caterpillar weights were recorded once every other day until they pupated to assess the mass gain.

### 2.9. Mass Gain

The equation below was used to calculate mass gain. For the lab experiment, we calculated two types of mass gains (i) mass gain over previous mass gains to determine variation in mass gain per day and (ii) mass gain over initial mass to determine mass gain relative to starting mass of the caterpillar, for similar studies see [24,26,27].

$$\mathrm{Mass}\mathrm{gain}\;=\;\frac{\left(\mathrm{Final}\mathrm{mass}\;-\;\mathrm{Initial}\mathrm{mass}\right)}{\left(\mathrm{Initial}\mathrm{mass}\right)}$$

### 2.10. Time to Pupate

Once the larvae reached the fifth instar, they stop feeding and begin to wander in the petri dishes (this stage is recognizable by morphological features such as a reddish-brown head and brownish body, which develops white subdorsal and lateral lines). At this point, the larvae were transferred to a plastic container containing wood shavings to provide a dark environment so that the larvae could undergo pupation. Pupation time was determined for each caterpillar by counting from the date they were placed on treatment diets to when they pupated.

### 2.11. Pupal Mass and Pupal Length

After the caterpillars pupated successfully (2–3 days after placing them in wood shavings), pupal mass was assessed by weighing each pupal from the field and laboratory experiments using a weighing balance (Accuris Dx W3101A-220, Valley Park, MO, USA). Pupal length was assessed with the aid of a Vernier caliper.

### 2.12. Polyphenol Oxidase (PPO) Assay

To test whether the cash crop defenses (from both cover grown and control fields) were affected, we estimated the activity of polyphenol oxidase (PPO). PPO is a widely distributed enzyme that plays a vital role in plant defense against plant diseases and herbivores [28,29]. Quantification of PPO was performed using the equation in the Polyphenol Oxidase Assay Kit manual (Catalog# MBS822343; MyBioSource, San Diego, CA, USA). PPO content (U/mg) was quantified in accordance with the methods described in the PPO Assay kit manual (Catalog# MBS822343; MyBioSource).

PPO (U/g) = (ODSample − ODControl) × VTotal/(W × VSample/VAssay)/0.01/T = 233.3 × (ODSample − 182 ODControl)/W

### 2.13. Scanning Electron Microscopy

A desktop Scanning Electron Microscope (DSEM; SNE-4500 Plus Table; Nanoimages LLC, Pleasanton, CA, USA) was used to observe the adaxial surface of sorghum plants planted either in the control or cover crop plots. Sorghum leaves (25 mm) were excised and fixed to a double-sided carbon tape that had been glued to an aluminum stub (40 mm). The aluminum stub, together with the leaf samples, was inserted into the DSEM, and images were viewed at 200× [30]. For comprehensive details about sample preparation and operational procedure of the DSEM, see [29,30].

### 2.14. Epicuticular Wax

Leaf epicuticular waxes were quantified from sorghum leaves planted in control and cover crop plots. Sixteen-hole punches were collected from sorghum leaves (three plants in each treatment across three fields) using a hole puncher. These leaf punches were placed in a preweighed 3 mL microcentrifuge tube containing 1.5 mL chloroform. These tubes were vortexed for 1.5 min, after which the leaf punches were removed. The chloroform was allowed to evaporate, after which the microcentrifuge tubes were reweighed to quantify epicuticular wax (mg) [28,31].

### 2.15. Statistical Analysis

Because some of our data did not meet the assumptions for normality, we used a combination of nonparametric tests (Mann–Whitney U), and *t* tests. Treatments were used as factors for field and laboratory experiments, while mass gain, pupation time, pupal mass, and pupal length, PPO (g), and epicuticular wax (mg) were used as response variables. Although fields were a significant factor in this experiment, the data from all the fields were pooled together to represent control and cover crop treatments, as we were more interested in seeing the effects of cover crops as a factor on the subsequent cash crop defense. Analysis showing the effects of the field as a factor is detailed in Supplementary Table S1. We also carried out a separate analysis for caterpillar mass gains at various stages of their growth and development for laboratory experiments. All analyses were carried out using SAS JMP (JMP^®^, Version <15>. SAS Institute Inc., Cary, NC, USA, 1989–2021), and plots were built using GraphPad Prism (GraphPad Software, version 9.0.0 for Windows, La Jolla, CA, USA).

## 3. Results

### 3.1. Mass Gains by Caterpillars from the Field Experiment

**Figure 1:**
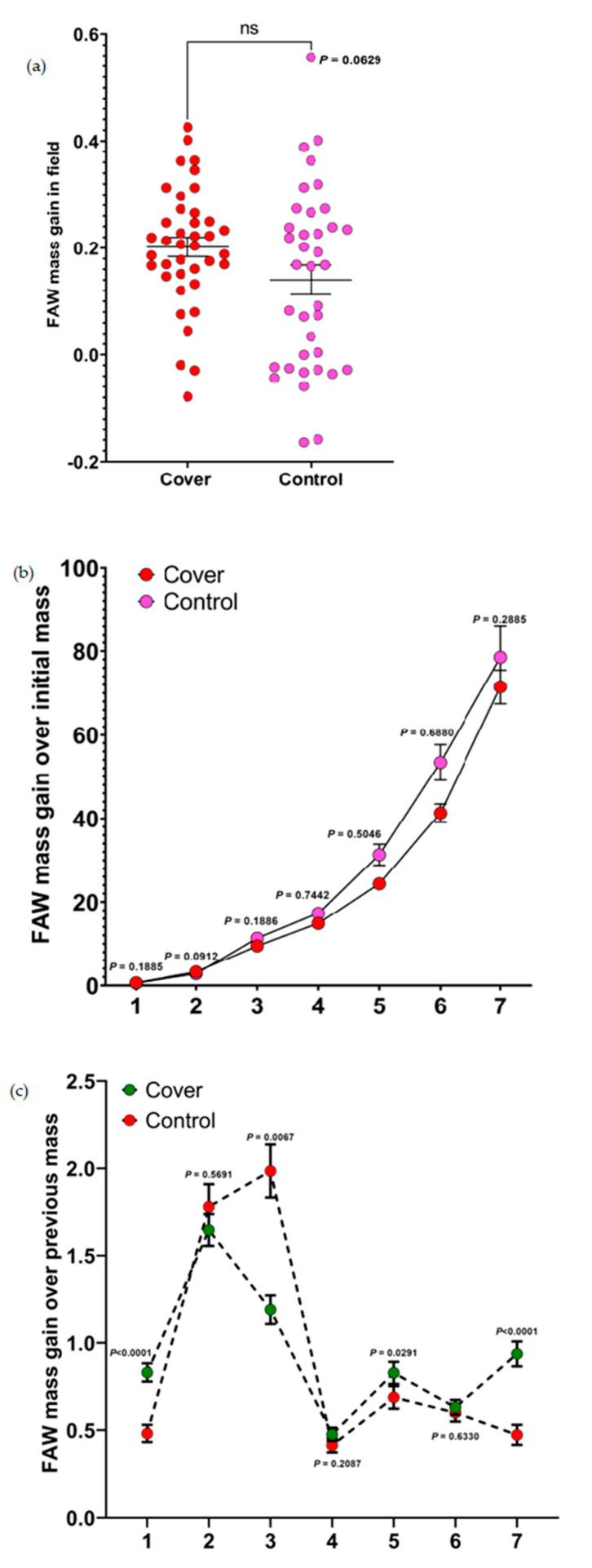
Results of the effect of control and cover crop treatment on the mass gain of FAW. (**a**–**c**) Mean mass gain of FAW comprising (**a**) effect of sorghum leaves planted in control and cover crop treatment plots on the mass gain of FAW (field experiment). Data were analyzed using *t*-tests (ns = not significant). (**b**) Effects of sorghum leaves planted in the control and cover crop plots on the mass gain of FAW (mass gain over initial mass). Individual mass gains were analyzed using Mann–Whitney *U* test (**c**) Effects of sorghum leaves planted in the control and cover crop plots on the mass gain of FAW (mass gain over previous mass). Individual mass gains were analyzed using *t*-tests.

For mass gains of caterpillars from the field experiment, the result from the paired *t*-test showed that the cascading effects of cover crops did not significantly impact them (*p* = 0.0629, Figure 1a). Although not significantly different, the caterpillars that fed on sorghum leaves planted in cover crop treatment plots had a higher mass than the caterpillars allowed to feed on sorghum leaves planted in the control plots.

### 3.2. Mass Gains by Caterpillars from the Laboratory Experiment

From the laboratory experiments, we computed seven mass gains (mass gain over initial mass and mass gain over previous mass). For mass gains over initial mass, there was no significant difference in the mass gains of caterpillars when placed on diets mixed with sorghum leaves from both control and cover crop plots (some data points showed 0 cm because some FAW caterpillars did not show any increase in mass gain over time) (Figure 1b). However, for mass gains over previous mass, we noticed that caterpillars fed on diets mixed with sorghum leaves from the cover crop treatments had significantly higher mass gains than caterpillars fed on diets mixed with sorghum leaves from the control plots (Figure 1c). This shows that cover crops had a positive effect on the caterpillars fed on diets mixed with sorghum leaves from the cover crop plots.

### 3.3. Pupation Time

**Figure 2:**
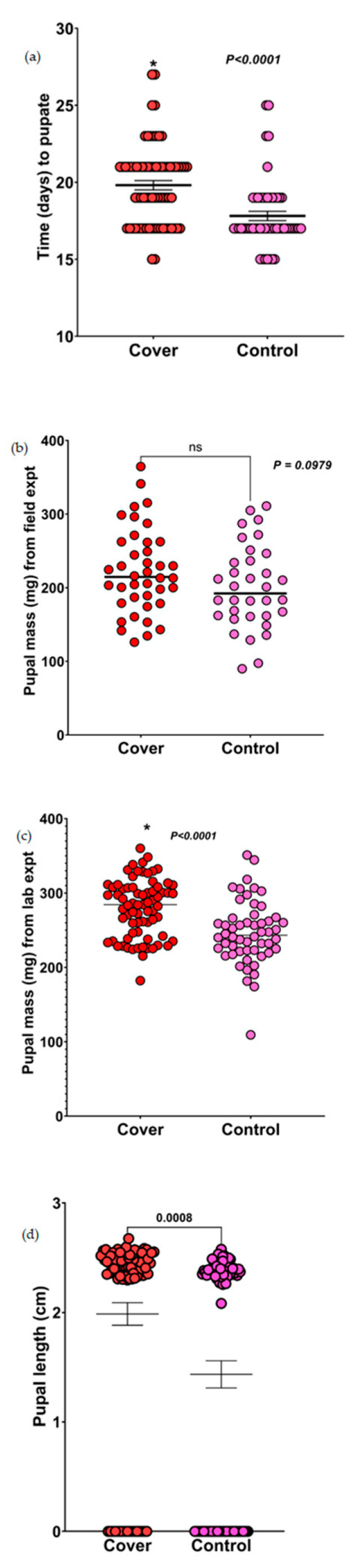
Results of the effect of the control and cover crop treatments on the pupal parameters of FAW. (**a**) Effect of sorghum leaves planted in the control and cover crop plots on pupation time of FAW (lab experiment). Data were analyzed using Mann–Whitney U test. (**b**) Effects of sorghum leaves planted in the control and cover crop plots on the pupal mass of FAW (field experiment). (**c**) Effects of sorghum leaves planted in the control and cover crop plots on the pupal mass of FAW (lab experiment). Data were analyzed using Mann–Whitney U test. (**d**) Effects of sorghum leaves planted in the control and cover crop plots on the pupal length of FAW. Data were analyzed using *t*-tests (ns = not significant and * denotes significance).

For the field experiment, both sets of caterpillars used a similar number of days to pupate. However, for the lab experiments, caterpillars fed on diets mixed with sorghum leaves planted in the cover crop treatments took significantly longer to pupate than those fed on diets mixed with sorghum leaves planted in the control plots (*p* < 0.0001; Figure 2a). This was interesting to see, since the FAW caterpillars fed on diets mixed with sorghum leaves planted in the cover crop plots had more mass gain, but also took longer to eclose.

### 3.4. Pupal Mass

For pupal mass from the field experiments, results from Mann–Whitney U tests showed no significant difference in the pupal mass of caterpillars fed on diets mixed with sorghum leaves from the control or cover crop plots (*p* = 0.0979; Figure 2b). Although not significantly different, the pupal mass of caterpillars fed on diets mixed with sorghum leaves planted in the cover crop plots was higher than that of caterpillars fed on diets mixed with sorghum leaves planted in the control plots. In the laboratory experiments, the pupal mass of caterpillars fed on diets mixed with sorghum leaves planted in the cover crop treatment plots was significantly higher than the pupal mass of caterpillars fed on diets mixed with sorghum leaves planted in the control plots (*p* < 0.0001; Figure 2c). This shows that the cascading effects of cover crops on the caterpillars translated into the pupal stage, although more pronounced in the lab assays under controlled conditions.

### 3.5. Pupal Length

The pupal length of caterpillars fed on diets mixed with sorghum leaves planted in the cover crop plots were significantly longer (*p* = 0.0008; Figure 2d) than caterpillars fed on diets mixed with sorghum leaves grown in the control plots (some data points showed 0 cm because we included the data of caterpillars that failed to pupate as 0). Collectively, we show that FAW caterpillars and pupae does better on cash crop from cover crop planted plots when examined in detail under controlled conditions in a lab.

### 3.6. Quantification of Polyphenol Oxidase (PPO)

**Figure 3:**
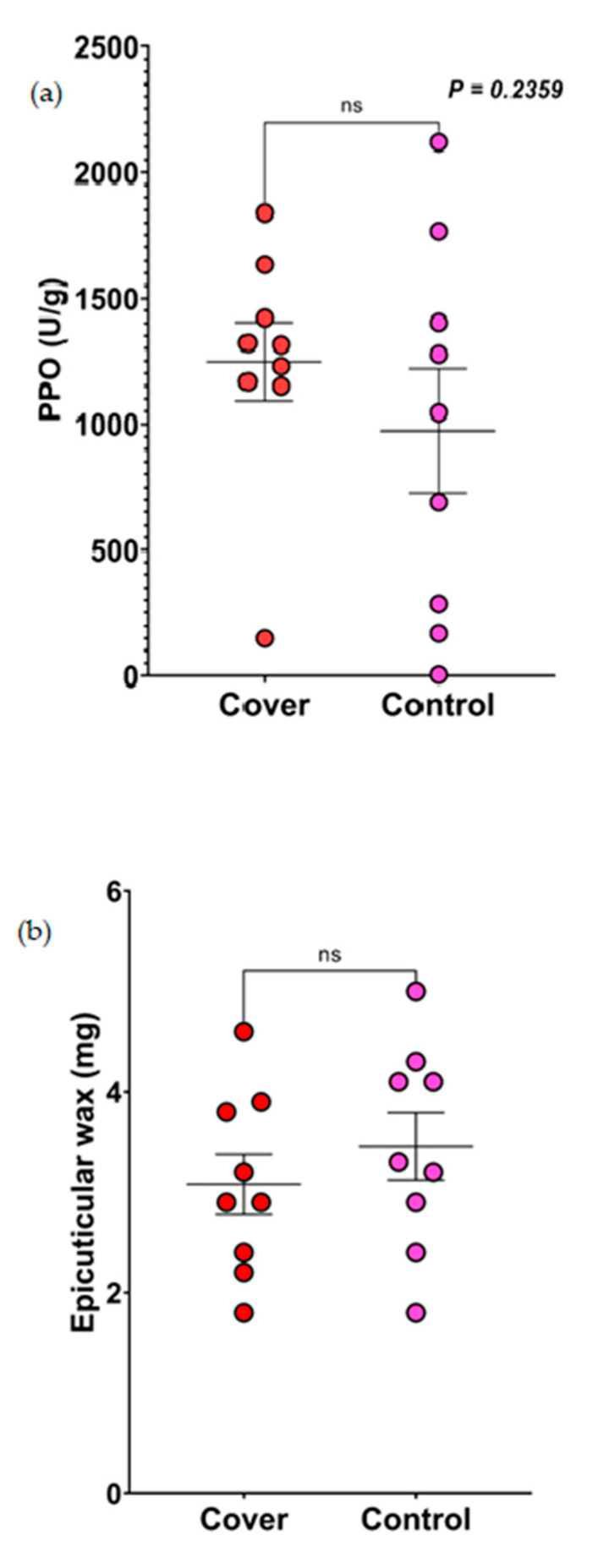
Results of the effect of control and cover crop treatment on the chemical and physical defenses in sorghum leaves. (**a**) Results of the quantification of PPO from sorghum leaves planted in the control and cover crop plots. Data were analyzed using Mann–Whitney U test. (**b**) Results of the quantification of epicuticular wax from sorghum leaves planted in the control and cover crop plots. Data were analyzed using *t*-tests (ns = not significant).

While quantifying PPO in the sorghum leaves from both the control and cover crop treatment plots across the fields, we found no significant differences (*p* = 0.2359; Figure 3a).

### 3.7. Epicuticular Wax

There was no significant difference in the wax amount in sorghum leaves that were either planted in the control or cover crop plots (*p* = 0.4145; Figure 3b). Also, there were no actual differences in the scanning electron microscope (SEM) surface images of sorghum leaves planted in the control or cover crop plots (Supplementary Figure S2a,b).

## 4. Discussion

This study was conducted to assess any possible cascading effects of cover crops on the growth and development of *S. frugiperda* in the subsequent cash crops across three fields in the Lower Rio Grande Valley (LRGV). FAW being a major herbivore, especially in the cash crop season, warranted a study that examined any possible effects that could affect its growth and development. Through field studies and an extensive and rigorous laboratory assessment, our results show that cover crops have cascading effects on the development of *S. frugiperda* in the subsequent cash crops, adding a line of evidence on how cover crops can affect pest dynamics outside the cover crop.

Recent studies have shown that the management of plant–soil feedback through cover cropping can result in positive, negative, or neutral feedback. The responses of plants to soil feedback can impact the growth and development of herbivores [11,32]. Additionally, cover crops such as legumes and grasses enhance the proliferation and colonization of arbuscular mycorrhizal fungi (AMF) [3,5]. These beneficial microbes have been reported to influence the regulation of plant defense against herbivores in subsequent cash crops [11,13].

We anticipated that caterpillars fed plant leaves and caterpillars fed on diets mixed with sorghum leaves planted in the cover crop treatments would have a significantly lower mass gain due to possible plant–soil feedback [3]. However, we observed that they had significantly higher mass gains than the caterpillars fed on sorghum leaves and the caterpillars fed on diets mixed with sorghum leaves from the control plots. A possible explanation for this outcome is that as much as AMF upregulates defense in plants against herbivores, they also enhance nutrient acquisition in plants, which could increase the attractiveness of cash crops to herbivores [11,33]. However, we did not estimate the AMF colonization success in cash crop, although this has been previously documented [3]. The direct and indirect effect of AMF on the alteration of plant defense against herbivores through the production of secondary metabolites, volatile organic compounds, and phytohormones has been enumerated in previous studies [3,11,34]. For example, [3] showed that plants grown with AMF received lower levels of herbivory compared to plants without AMF. Also, [34] showed that AMF upregulated the defense in maize plants against herbivores by enhancing the expression of defense-related genes (*mpi* and *pr5*).

We quantified PPO content in sorghum leaves grown in control and cover treatment plots to evaluate the cascading effects of cover crops on the subsequent cash crop defense against FAW. Caterpillars fed on sorghum leaves and caterpillars fed on diets mixed with sorghum leaves planted in the cover crop plots had a significantly higher mass gain, suggesting that PPO, and possibly other plant defenses, were not enhanced in cash crop, as previously suggested by [11,13].

A suite of previous studies has shown that plants employ both chemical and physical defenses against herbivores [25,28,35]. Although most studies have focused on how plants elicit chemical defense compounds to deter herbivores, some studies have also shown that physical traits such as trichomes and epicuticular wax impact herbivore feeding [28,36]. We quantified the wax amounts on sorghum leaves planted either in control or cover crop plots to determine if epicuticular wax impacts the feeding of FAW. We found no significant differences in the amount of epicuticular waxes in sorghum leaves planted in the control and cover crop plots across the fields. Although not significantly different, there were higher amounts of epicuticular wax in the sorghum leaves planted in the control plots. This, however, might be a probable reason why FAW had higher mass gains when fed on sorghum leaves planted in the cover crop plots [28,36,37].

Past studies have evaluated the differential effects of different diets on FAW’s survival and mass gains and if these effects cascade through their growth and development [24,38,39]. These showed diet-dependent differences for larval mass gains, pupal weight, development time, and mortality. It was not surprising to see that the pupal mass of caterpillars that fed on diets mixed with sorghum leaves planted in the cover crops plot had significantly higher mass than the caterpillars that fed on diets mixed with sorghum leaves planted in the control plots. This can be attributed to the fact that the effects of cover crop treatments on FAW in the larval stage translated into the pupal stage. Furthermore, the pupal length of caterpillars fed on diets mixed with sorghum leaves planted in the cover crop plots was significantly higher than that of caterpillars fed on control diets. Even though predation risk can be high during the pupal stage because it is the immobile developmental stage, studies have shown that they are not defenseless, as they have evolved strategies against predators [40,41]. Although not significant, we noticed a similar trend with the field experiments. This shows that the effects of the diets fed to the caterpillars cascaded through the larval and pupal stages. It should also be noted that the field study only allowed us a snapshot of FAW growth and development, since we could only allow them to feed for 3 days in the field (imminent storm). The lab data also suggest that a snapshot of field feeding may miss other effects unless long-term feeding assays or enriched diet assays that go beyond mass gain [42] are carried out to test the effects of both physical and chemical defenses.

## 5. Conclusions

In conclusion, we show that FAW, a devastating polyphagous pest, can be differentially impacted by cover–cash crop rotations. Furthermore, our laboratory and field results show that cover crops had a possible cascading effect on the defense of the cash crops against FAW, which was revealed through pupation time, mass gains, pupal mass, and pupal length. However, since our constitutive defenses experiment failed to show any differences, detailed exploration of other possible mechanisms should be done to predict how these cascading effects lead to trophic interactions in agroecosystems, a key consideration for sustainable agriculture.

## Figures and Tables

**Table 1:** Cover crop treatments, species with their respective seeding rates.

Field	Cover Crop Treatments	Crop Type	Seeding Rate (kg/ha)
Hunter	Sorghum sudangrass and cowpea	Grass and Legume	2.3 + 5.4
Cemetery	Cowpea, sun hemp, and sorghum–sudangrass	Legume and Grass	5.4 + 5.4 + 2.3
Mahac	Cowpea and radish	Legume and vegetable	6.8

**Table 2:** Cash crops, species with their respective seeding rates.

Field	Cash Crop	Crop Type	Seeding Rate (kg/ha)
Hunter	Sorghum	Grass	2.3
Cemetery	Sorghum	Grass	2.3
Mahac	Sorghum	Grass	2.3

## Data Availability

All raw data will be made available on reasonable request.

## Supplementary Materials

The following supporting information can be downloaded at. https://www.mdpi.com/article/10.3390/insects14020177/s1. Figure S1: Regional map and individual fields used in the study; Figure S2: Electron micrograph (200×) of the adaxial side of sorghum leaves planted in the control and cover crop plots; Table S1: Results of the statistical analyses to examine the effects of cover crops on the subsequent cash crop defense against FAW.
