# Supplementary material for: Cascading Effects of Cover Crops on the Subsequent Cash Crop Defense against the Polyphagous Herbivore Fall Armyworm (Spodoptera frugiperda)
Source: Insects. 2023 Feb 10;14(2):177. doi: 10.3390/insects14020177 (PMC9959575; doi:10.3390/insects14020177)
Supplement: Supplementary file 1 [file insects-14-00177-s001.zip › insects-2190309-supplementary.pdf]

Table S1. Results of the statistical analyses to examine the effects of cover crops on the subsequent cash crop defense against fall armyworm

Experiment	Traits	Test Statistic	P value
Field experiments	Mass gain (Treatment)	F = 1.04, df = 1	0.3110
	Mass gain (Field)	F = 10.35, df = 2	0.0001
	Mass gain (Treatment * Field)	F = 0.64, df = 2	0.5314
	Pupal weight (Treatment)	F = 2.70, df = 1	0.1046
	Pupal weight (Field)	F = 16.94, df = 2	<0.0001
	Pupal weight (Treatment * Field)	F = 1.04, df = 2	0.3588
	Days to pupate (Treatment)	F = 0.04, df = 1	0.8367
	Days to pupate (Field)	F = 4.11, df = 2	0.0206
	Days to pupate (Treatment * Field)	F = 0.61, df = 2	0.5448
Lab experiments	Pupal mass (Treatment)	F = 29.65, df = 1	<0.0001
	Pupal mass (Field)	F = 31.11, df = 2	<0.0001
	Pupal mass (Treatment * Field)	F = 22.73, df = 2	<0.0001
	Days to pupate (Treatment)	F = 315.69, df = 1	<0.0001
	Days to pupate (Field)	F = 104.98, df = 2	<0.0001

Days to pupate (Treatment * Field)	F = 224.80, df = 2	<0.0001
Wax (Treatment)	F = 0.84, df = 1	0.3769
Wax (Field)	F = 2.54, df = 2	0.1165
Wax (Treatment * Field)	F = 1.01, df = 2	0.3931
PPO (Treatment)	F = 0.86, df = 1	0.3717
PPO (Field)	F = 0.02, df = 2	0.9731
PPO (Treatment * Field)	F = 1.76, df = 2	0.2137
Pupal length (Treatment)	F = 19.39, df = 1	<0.0001
Pupal length (Field)	F = 38.33, df = 2	<0.0001
Pupal length (Treatment * Field)	F = 23.17, df = 2	<0.0001

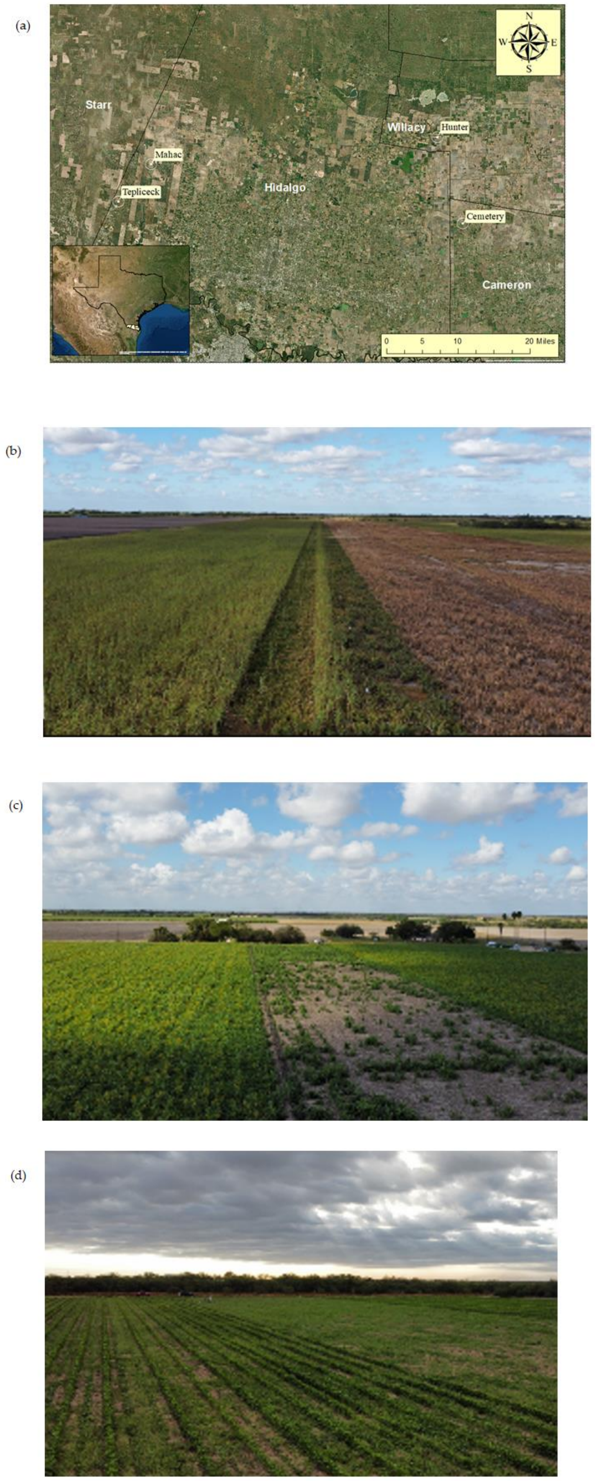

Figure S1. Regional map and individual fields used in the study. (a) Regional map of the fields, (b) Cemetery field showing control and cover crop plots during cover cropping season, (c) Mahac field showing the control and cover crop plots during cover cropping season, (d) Hunter field showing the control and cover crop plots during the cover cropping season.

(a)

(b)

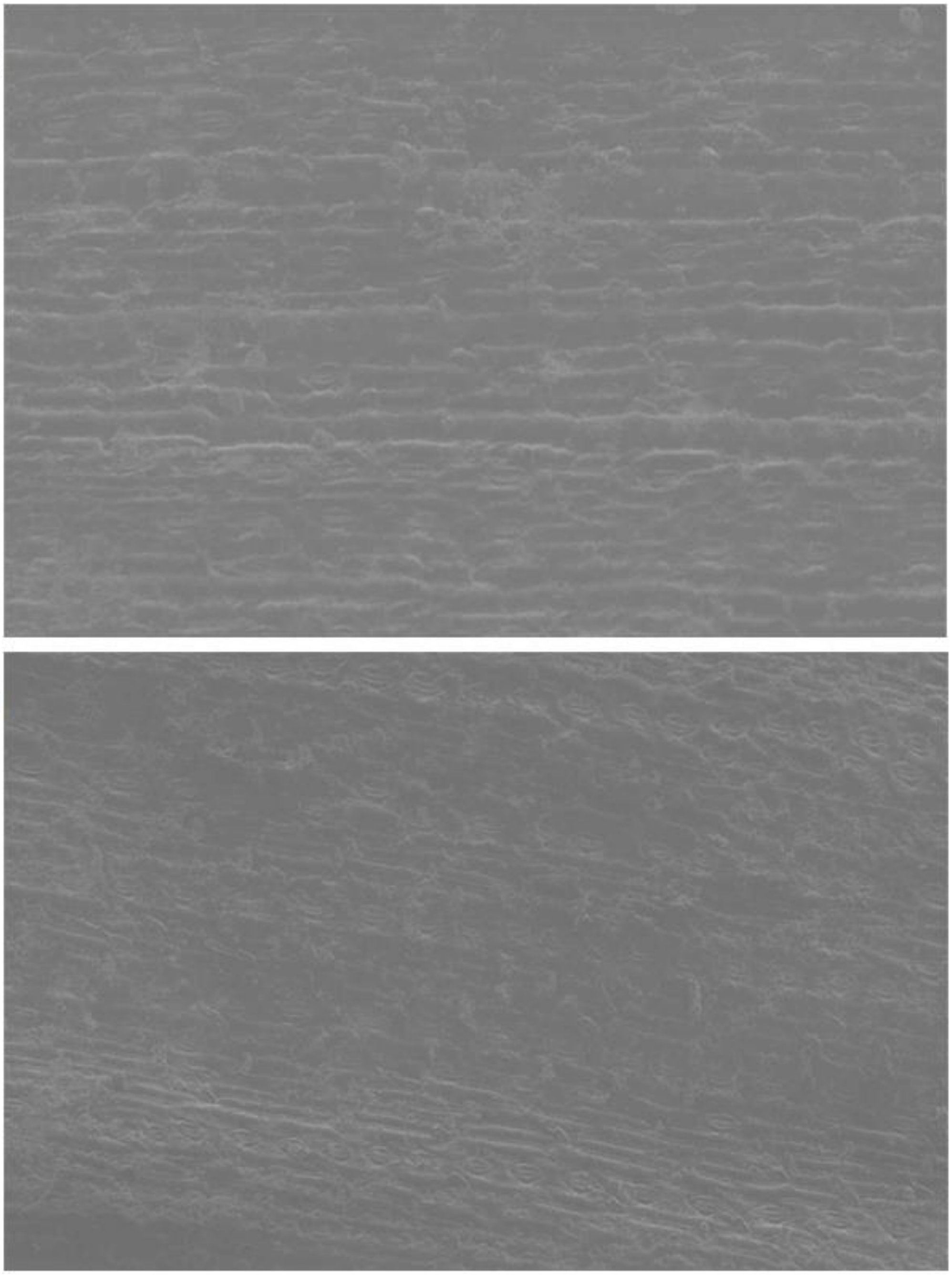

Figure S2. Electron micrograph (200X) of the adaxial side of sorghum leaves from both control and cover crop plots. (a) electron micrograph (200X) of the adaxial leaf surface of sorghum leaves planted in the cover crop treatment plots, (b) electron micrograph (200X) of the adaxial leaf surface of sorghum leaves planted in the control plots.
